# John Walford McLean – the dental innovator who led the British development of resin composites

**DOI:** 10.1038/s41415-026-9621-y

**Published:** 2026-08-28

**Authors:** Garry J. P. Fleming

**Affiliations:** Materials Science Unit, Dublin Dental University Hospital, Lincoln Place, Trinity College Dublin, Ireland

## Abstract

From publishing his first paper as a dental undergraduate in 1950 to the development of aluminous core porcelain in 1965, John Walford McLean was at the forefront of the British contribution to dental materials research. The ability to recognise material limitations at an early stage, combined with the confidence in his decision-making was what set John McLean apart from any dental innovator that had gone before – or arguably that came after! The British contribution to dental materials research was responsible for the introduction of reinforced dental porcelains, polyacrylic acid-base cements, glass-ionomer cements and the epoxy resin systems from 1943 to 1983 and John McLean, was at the forefront of these innovations. Depressingly in 1984, despite over 30 years at the coalface of dental materials development, McLean suggested gold and porcelain to be the two permanent dental materials available to the dental practitioner. In 1980, McLean predicted the future of aesthetic restorative dentistry lay in developing new technologies rather than the simple substitution of one material for another. He was not wrong!

## Key points

- A historical review of the early years of resin composite development with recognition of the participation and appraisal of the UK effort often omitted from the dental literature.
- A snapshot of the thinking and rationale of John McLean, expressed through his publications, in the quest of ‘preserving or even replacing, human tooth enamel'.
- McLean predicted the future of aesthetic restorative dentistry lay in developing new technologies rather than the simple substitution of filler (type, size and density) with minor alterations to the monomeric resin composition.

## Introduction

### The challenge

What is the ‘challenge' facing dentistry today? Everybody has an opinion, but in 1984, John McLean – the greatest dental innovator – provided rare insight from his perspective, entitled ‘The British contribution to dental materials research and its impact on the future course of treatment in restorative dentistry',^1^ saying:

‘*On a more optimistic note, one of my more stimulating experiences during 20 years' work with the government science laboratories is the appreciation and, indeed, respect that I have seen given by some of our most eminent scientists to the problem of preserving, or even replacing, human tooth enamel. To date, the development of alternative materials to those used for so long has baffled our best brains in the laboratories of the Government Chemist, the British Ceramic Research Association, the National Physical Laboratory and the Atomic Weapons Research Establishment, to name but a few who have addressed themselves to this problem*'.^1^

Over the previous 40 years (1943–1983), UK dental materials research was responsible for the introduction of reinforced porcelains,^2^ polyacrylic acid-base cements,^3^ glass-ionomer cements,^4^ and the epoxy resin systems.^5^ McLean^6^ acknowledged the work of Bowen in 1958^7^ that ‘finally led to the development of bis-glycidyl methacrylate resins which are now used in composite filling materials'.^6^ But what of those early years of resin composite development?

## John Walford McLean

As a final-year dental student in London during the Second World War, McLean used one of the first acrylic resins (Portex ‘Quick Set' Filcryl, developed for Portland Plastics of London by S. A. Leader in 1944) to make acrylic inlays. However, cemented with zinc phosphate they were susceptible to microleakage.^8^ McLean decided to manufacture an acrylic cement with a ‘faster' setting time and removed the hydroquinone inhibitor present in the monomer by distilling under vacuum, thereby, improving the shelf-life.^8^ With the addition of benzoyl peroxide as initiator, he used a low-viscosity mix of acrylic resin as cement for those inlays, but still slow setting meant that the margins ‘needed protection'.^8^ Then:

- ‘*Remembering that we processed our acrylic denture bases under pressure, I tried out the use of a rubber sleeve (cut from bicycle valve tubing) slipped over the inlay and the tooth during polymerisation to protect the margins and, I hoped, to apply some pressure. My first paper describing these techniques was published while I was a student.*^9^*From this beginning, my interest in making new materials never waned and led me to develop the “screw pressure crown”, an acrylic crown screwed to the root of a devitalised tooth with an acrylic monomer-polymer mix as the cement*^10^*…although the crowns remained firm, the wear of acrylic soon convinced me that these restorations were only for the short term. My interest in dental porcelain stemmed from this acrylic shortcoming*'.^8^

**Fig. 1:**
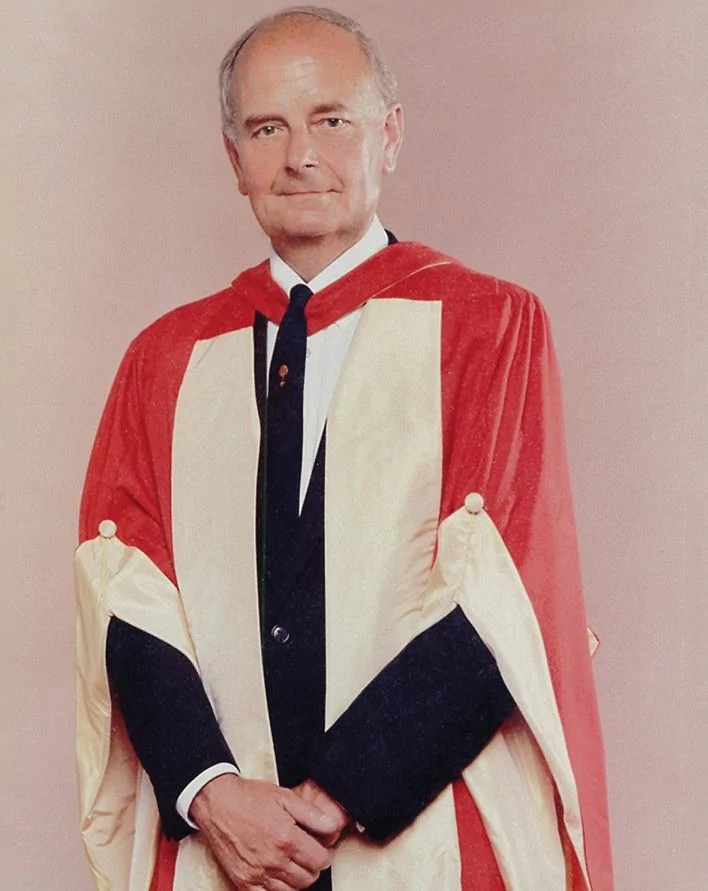
A picture of John McLean when he was awarded a Doctorate of Science (DSc) in 1978 by London University for his work on the advances in new dental materials and their physical and biological assessment. It was unique for a dentist still in practice to achieve these distinctions. For years afterwards. McLean spoke with pride of receiving his doctoral hood from Queen Elizabeth the Queen Mother on the last occasion she presented awards as Chancellor of the University (image courtesy of BDA Museum)

It was indeed this ability to recognise material limitations at an early stage, combined with the confidence in his decision making in abandoning an idea in favour of another – despite the obvious advancements made, was what set John McLean (Fig. 1) apart from any dental innovator that had gone before – or arguably that came after!

## The early years of resin composite development

**Fig. 2:**
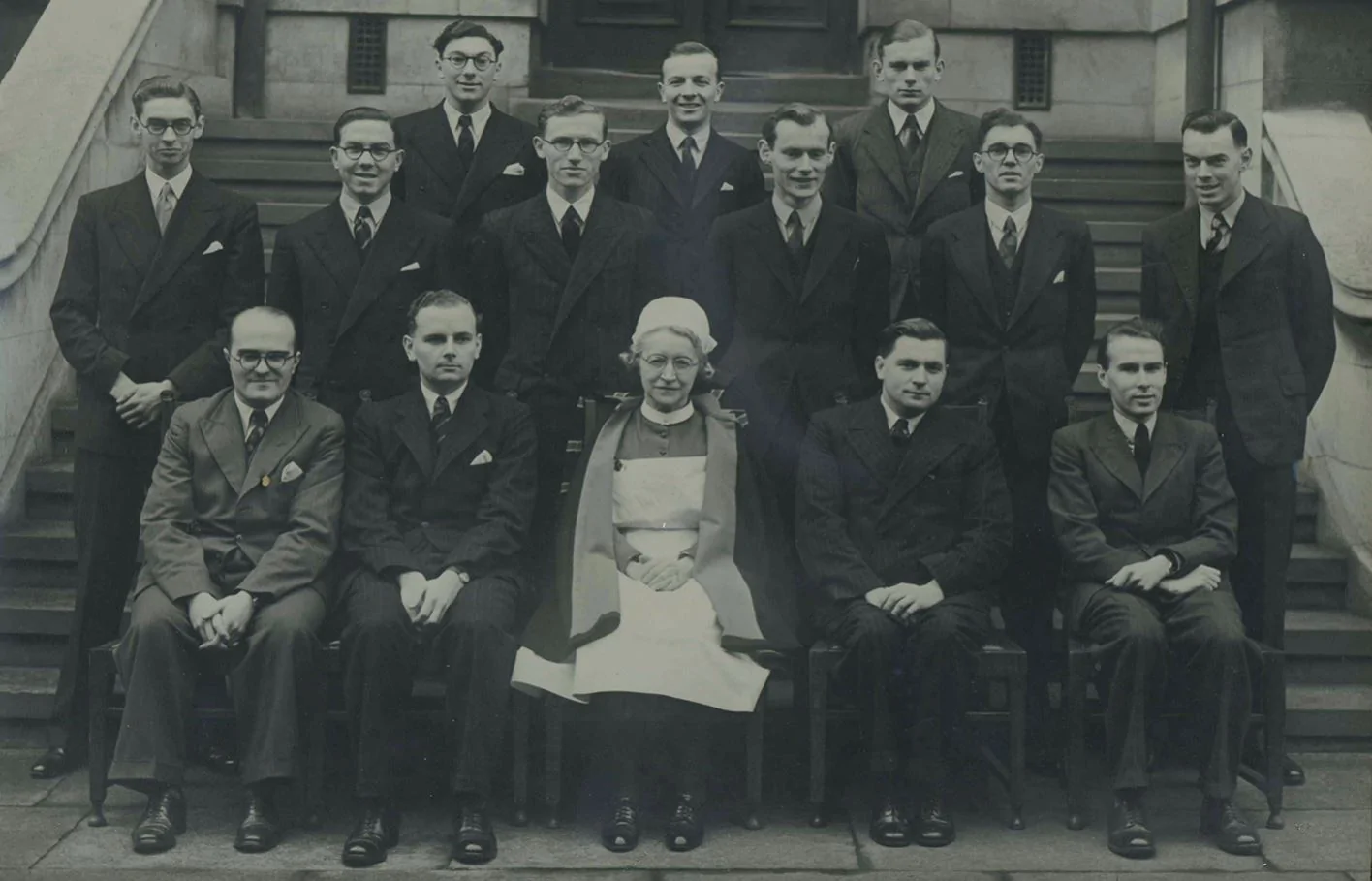
A group photo with John McLean (front row, seated, second from left) as a house officer at Guys in 1947 (image courtesy of BDA Museum)

Documented details of a 1947 German technical report,^11^ into a cold-curing acrylic resin developed for restorative dentistry, provided a milestone in dentistry, namely the first description of the tertiary amine-benzoyl peroxide system.^12^ While ‘Filcryl' was revolutionary in 1944, high polymerisation shrinkage, wear, discoloration and leakage,^13^ on top of the ‘slow initial and finite polymerisation',^14^ were major problems resulting in dentists ‘returning to the use of silicate cements'.^13^ McLean (Fig. 2) published an investigation into the physical properties, histopathology and clinical technique of the mouth-temperature, cold-curing acrylic resins in 1950 with the encouragement of ‘our most distinguished pathology teacher', Professor Martin Rushton.^14^ There was ‘considerable damage to the odontoblasts' under ‘Filcryl', but of more interest was the ‘aspiration of odontoblasts into the dentine tubules and the subsequent formation of oedema blisters in the pulp'^14^ due to the free monomer present. McLean concluded that ‘it would seem advisable to line all cavities until the safety of these new resins has been established'.^14^ In the absence of a liner material, the self-polymerising resins were capable ‘of causing polymorphonuclear infiltration, generalised cellular infiltration of the pulp, secondary dentine formation and aspiration of the odontoblasts'.^15^

Hagger^16^ is credited with the development of sulphinic acid catalysts which led to improved colour stability and shelf-life of acrylic restoratives. Focusing on the problem of microleakage at the cavity margins resulted in the development of the first coupling agents which ‘promoted adhesion of the tooth surface to the restorative'.^16^ The Hagger material was marketed as Sevriton (Amalgamated Dental Co., England) and was employed in conjunction with a coupling agent based on glycerophosphoric acid dimethacrylate, namely, Sevriton Cavity Seal. Patented on 15 November 1951,^17^ this was the first adhesive to use a form of acid-etching.^12^ Hagger collaborated with McLean^18^ during the development of the Sevriton bonding system and Kramer and McLean^19^ used glycerophosphoric acid dimethacrylate to bond Sevriton to enamel and dentine in prepared cavities in non-carious human teeth. Sections from 124 cavities in 118 teeth, filled with 15 material/techniques, highlighted an alteration to the staining reaction of the dentine in all 28 teeth filled with Sevriton and Sevriton Cavity Seal that were not evident in the 96 other filled cavities employed'.^19^ This was the first time in the dental literature that an adhesive filling material was studied. The ‘efficacy of the Sevriton cavity adhesive' was further demonstrated,^20^ indicating that glycerophosphoric acid adhesion was by penetrating the dentinal surface, forming an intermediate layer today called the hybrid zone. While most researchers quote Michael Buonocore's 1955 manuscript^21^ as the defining study in dental adhesion, the statement is incorrect.^18^ Buonocore did use a stronger acid (i.e., phosphoric) to etch the enamel, which has been interpreted historically as opening the way for adhesive restorative dentistry. However, in 1958^22^ Buonocore had used Sevriton in a histological study of the bonded area, referencing Kramer and McLean's 1952 paper,^19^ which authors therefore ultimately deserve the credit, along with Castan and Hagger, who ‘set us upon the path of developing chemically adhesive restoratives'.^18^

McLean reported the results of a clinical study initiated in 1957 on an experimental acrylic-based material (TB71, Dental Fillings Ltd., UK), composed of a 60 mass% silicate glass powder and a 40 mass% acrylic polymer powder in an attempt to improve the marginal seal and abrasion resistance of acrylic-based restorative materials.^23^ The clinical evaluation techniques for TB71 and the silicate cement control were based on the results of both visual and microscopic studies in accordance with the Hedegard's 1955 criteria:^13^

- Good – no objection can be taken to fit
- Fair – marginal accuracy clinically acceptable but traces of strain noticeable at the margins of the filling
- Unsatisfactory – distinct discolouration at the joint which can be clearly felt with a probe
- Bad – strong discolouration at the edges of the cavity where a sharp probe will catch the joint.

However, following clinical evaluation the deficiency of the experimental acrylic-based material (TB71) was related to ‘inadequate bonding of filler and binder resin'.^24^ From the inception of the clinical study in 1957, to the publication of the less than favourable results in 1961,^24^ there was been a movement towards ‘aromatic dimethacrylates over the old acrylic resins (methylmethacrylate)'.^12^ Dr Robert Purrmann and Eric Schmidt founded the ESPE Company and ‘produced the first low viscosity resin based on the Epimine resin patent filed in June 1958';^8^ the resulting ESPE composite (P-Cadurit) contained glass beads as a filler. In an attempt to further improve the properties of acrylics (increased elastic modulus and abrasion resistance and decreased polymerisation shrinkage), Cadurit (ESPE Fabrik Pharmazeutischer Praparate, Seefeld, West Germany), was introduced as an anterior filing material. Cadurit was a glass-filled aziridino polyester with a sulfonic acid ester added to initiate polymerisation with the manufacturers claiming that the set filling material had ‘no water soluble components and no monomers,' when marketed.^3^ Indeed, in 1961, McLean compared the new crosslinked Cadurit material with a direct acrylic filling material in terms of the physical properties defined as dimensional changes on polymerisation, coefficient of linear expansion, surface hardness, compressive strength, water absorption and marginal percolation.^14^ Unfortunately, there were no apparent advantages in the physical properties of Cadurit compared with the direct acrylic filling material investigated.^14^

In the United States, aromatic dimethacrylates can be traced back to a Progress Report^7^ by Bowen on the ‘Synthesis of a silica-resin direct filling material' in the *Journal of Dental Research* in 1958. The report was based on the ‘reaction product of bisphenol A and glycidyl methacrylate, thinned with tetraethyleneglycol dimethacrylate and activated with dimethyl-para-toluidene, hardened at room temperature in about three minutes when mixed with fused silica containing benzol peroxide'.^7^ Further investigation and development of the material focused initially on the tensile strength and stiffness,^25^^,^^26^ and five years after the original Progress Report,^7^ the incorporation of the vinyl silane-treated silica powder (0.5 mass% silane) into the organic binder [primarily the reaction product of bis(4-hydroxyphenyl) dimethylmethane and glycidyl methacrylate catalysed by 0.5% N,N-dimethyl-p-toluidene], confirmed the reinforcement of the resultant material.^26^ The silica-reinforced polymer had ‘less brittleness than silicate cements', and was suggested as ‘an aesthetic and permanent restorative material that could be placed directly into the cavities prepared in anterior teeth',^26^ leading to the development and marketing of a new anterior resin-composite filling material (Addent 35, Minnesota, Mining and Manufacturing, USA).^27^

In January 1963, McLean presented a paper on mineral fillers for use in dental composite materials to the Department of Scientific and Industrial Research in London, saying ‘*I was looking for fillers with low specific gravity, a refractive index matching that of the resins used, low absorption power, and a mean statistical diameter of three to five microns which were readily dispersible in the resin without causing agglomeration of the particles*'.^8^ The chief experimental officer at the Warren Spring Laboratory at Stevenage, England was T. Harry Hughes, who suggested a number of materials including a sintered alumina that ultimately led to the development of aluminous core porcelain^2^ and the birth of aesthetic restorative dentistry to replace metal-ceramic restorations. Dr Dennis Smith – who discovered polycarboxylate cements from the ‘concept of producing chemically adhesive materials'^8^ – also worked in 1968 on high-temperature polymer coating of inorganic fillers. Dental Fillings Ltd., UK, produced the first commercial material based on the high-temperature polymer-coated fillers, namely TD71, using a fluoride-leachable alumino-silicate glass based on the pioneering work of Shadbolt at Dental Fillings Ltd., which overcame the problems of ‘inadequate bonding of filler and binder resin' in TB71.^24^ High-temperature polymer coating of the fillers was advantageous in that the filler could be freshly primed with silane, immediately coated in polymer, thereby improving the filler to resin bond integrity.^24^ A clinical and physical appraisal of anterior composite filling materials, namely acrylic polymer with inorganic filler (TB71), liquid resin with inorganic filler (Cadurit and Addent 35) and liquid monomer with polymer coated inorganic filler (TD71), were compared with a silicate cement and published in 1969.^28^ The acrylic- and aromatic dimethacrylate-based anterior filling materials^28^ were assessed clinically for colour stability, surface contour, marginal discolouration or deep discolouration by visual examination.^13^^,^^23^ TB71 fillings evaluated at three years^23^ had a significant loss of surface contour with unacceptable marginal discoloration which was exacerbated after seven years observation.^28^ These results precluded their use clinically owing to the decreased performance compared with the silicate cement control.^28^ The three year results for Cadurit were poor, compounded by a colour change for all restorations from a light yellow to a dark brown, loss of surface contour owing to plucking of the glass beads and total breakdown at the resin-filler interface of Cadurit, possibly owing to hydrolylic instability.^28^ Addent 35 restorations initially suffered from poor colour stability after two years; however, loss of surface contour was not significant, with limited marginal discoloration evident.^28^ While overall the results were positive compared with Cadurit, the entrapment of air bubbles in Addent 35 restorations and the viscosity of the aromatic dimethacrylate-based Addent 35 was in the authors' words ‘a little sticky'.^28^ High-temperature polymer coating of the fillers in TD71 was advantageous in improving the filler to resin bond integrity and clinical performance was improved compared with TB71 and silicate cement restorations but not compared with respect to Addent 35.^28^ Over the next five years an explosion of resin-based composite materials were introduced into the dental market. The majority were based on Bowen's bis-GMA-TEGDMA resin owing to the advantages of increased strength, modulus of elasticity, hardness and resistance to abrasion, combined with reduced polymerisation shrinkage and coefficient of thermal expansion compared with conventional acrylic resins.^29^^,^^30^ This was confirmed in 1975 by Asmussen using nuclear magnetic resonance analysis: 23 of 26 restorative resins were based on Bowen's resin, of the remaining three resins, one had methacrylic acid (Addent 12 cat, 3M, USA) and the remaining two (TD71 and Sevriton) had methyl methacrylate as the main monomeric resin constituent.^31^

## Regulation

In 1971, the Council on Dental Materials and Devices (USA) reported on the recommended uses of ‘composite restorative materials'^32^ where a ‘composite' indicated the ‘presence of reinforcing fillers such as glass rods and beads, aluminium silicate, quartz, and tricalcium phosphate which are surface treated to bond to a resin that serves as the matrix material'.^32^ After reviewing the advertising copy and promotional brochures for the early composite materials (Adaptic, Johnson & Johnson, USA; Addent; Blendent, Kerr, USA; Concise, Minnesota, Mining and Manufacturing, USA; Dakor, Caulk Division of Dentsply International, USA; DFR, Surgident, USA; Epoxylite HL 72, Lee Pharmaceuticals, USA; Posite, American Consolidate, USA; TD 71, Dental Fillings, UK), the Council recognised the principal effectiveness of these materials to be in Class III and Class V restorations,^32^ acknowledging that the differences in the physical properties between materials ‘does not appear significant from a clinical standpoint'.^32^ However, the Council also reported that ‘the exact overall role of composite material still awaits the outcome of well-controlled clinical investigations' and therefore composite product selection for practitioners should be made ‘objectively and be cautious in regard to claims made for the various composite resins'^32^ until additional information became available.

The ‘additional information' came in the form of well-controlled clinical investigations initiated by Ralph Phillips and co-workers^33^^,^^34^^,^^35^ who compared Adaptic with a representative amalgam alloy (Velvalloy, SS White Dental Products, USA) for Class II restorations in bicuspids and molar teeth. ‘Adaptic' was introduced to the market in 1969 as ‘Adaptic invisible fillings' which reportedly mirrored the properties of a healthy tooth. Baseline examination of 109 pairs of restorations was made within two months using a precursor to the soon-to-be-established United States Public Health Service (USPHS) technique reported by Cvar and Ryge.^36^ After one year, marginal failures occurred at a lower rate for Adaptic compared with the amalgam, although these were superior in terms of the maintenance of anatomic form.^33^ The frequency of marginal breakdown was similar for both at two^34^ and three^35^ years, but with changes in anatomical form evident in half the Adaptic fillings from occlusal wear at two years^34^ and further exacerbated at three years.^35^ It was concluded that until improvements to the wear resistance of the resin material was addressed ‘the routine use of composite resins in Class II restorations would seem to be contraindicated'.^35^

In 1975, four resin composites (Adaptic, Blendant, Concise and DFR), one acrylic resin (Sevriton, Amalgamated Dental Trade Distribution, UK) and a representative amalgam alloy (Velvalloy) were evaluated at two years using the USPHS technique^36^ for Class I, II, III and V cavity preparations.^37^ Leinfelder and co-workers^37^ reported composite wear was less than for acrylic, although in Class I and II cavity preparations was ‘appreciably greater' than for amalgam: two thirds (66%) of the 668 composite fillings had significant wear compared with one in eight (12%) of the amalgams.

In response to those reports,^33^^,^^34^^,^^35^^,^^37^ the Council on Dental Materials and Devices' ‘Certification and classification programs for dental materials and devices'^38^ provided a ‘List of Certified Dental Materials and Devices' revised to January 1977. For the first time composite filling materials were included as ‘Acceptable (provisionally acceptable) for use in Class III and Class IV restorations and for limited use in Class I restoration in premolars and selected Class IV restorations where aesthetics is of primary importance'.^38^ It is of note that the published findings stated that ‘the responsibility for providing and substantiation of claims for safety and efficacy or claims of compliance with an official standard shall reside with the manufacturer and not the American Dental Association'.^38^

In the subsequent follow-up, Leinfelder *et al.* reported on the five year clinical evaluation^39^ and concluded that the resin composite formulations ‘should be restricted to anterior teeth' since the occlusal and proximal wear in Class I and Class II cavities precluded their usage clinically.^39^ The material loss of posterior resin-composite restorations in the interproximal contact area was of concern since the rubbing of surfaces at the interproximal area stressed the protruding glass particles and led to them being plucked out, and this exacerbated wear in a manner similar to that occlusally.^39^ Consequently, there was reluctance among dental practitioners to employ this first generation of composite resins in Class I and Class II preparations, despite in many instances the perceived patient need for ‘aesthetic' restorative treatment.

Following a meeting in November 1980, the Council on Dental Materials, Instrumentation and Equipment (CDMIE) reported ‘that sufficient evidence has accumulated to include in the Acceptance Program those composite resin materials used for occlusal Class I and Class II restorations'.^40^ However, there was a sting in the tail:

- ‘A number of manufacturers and clinicians have advocated the use of composite resin materials in virtually all types of cavity preparations. In order to provide minimum requirements for physical characteristics, biological compatibility, and clinical effectiveness for these materials, guidelines have been established by the Council. For classification of a product under the Acceptance Program, adequate evidence must be presented to show that the composite material, when used in the posterior region of the mouth, is as effective as dental amalgam in regard to clinical service. This evidence must include at least two independent studies of at least three years' duration'.^40^

In 1983, reporting on the issue of the safety of dental amalgam, the CDMIE and the Council on Dental Therapeutics of the American Dental Association^41^ stated ‘except in individuals sensitive to mercury, there is no reason why a patient should seek to have amalgam restorations removed.' Again in 1983, the CDMIE^42^ stated that ‘an acceptable resin-based substitute for amalgam that can be used unrestrictedly as a restorative material for stress-bearing posterior restorations is not currently possible.' Ironically, 1983 also happened to be ‘the first time the dental literature has reflected the increased acceptance of commercial composite restoratives based on fillers of very different particle size and chemical composition'.^43^ A classification^44^ and evaluation of resin composite systems^45^ with reference to the particle size distribution of the fillers resulted in a ‘state of the art' classification of composites into macro fillers, micro fillers (homogeneous micro fillers and heterogeneous micro fillers) and hybrid composite resins. The patenting of visible light-curing initiators based on α-diketone-amine systems^46^ by Dart and Nemcek at Imperial Chemical Industries, Macclesfield, UK, further increased the popularity of these resin-bonded composites throughout 1984 and 1985,^47^ and this was reflected in a number of review articles highlighting their ‘current status'.^48^^,^^49^ Also, within this transformative time for tooth tissue-mimicking restorative dentistry, light-irradiated RBCs had ‘come of age'^50^ along with an increased understanding of the associated technical problems.^51^

## The state of play: 1981 to 1989

In December 1984, the proceedings of the first workshop on ‘The Introduction of New Materials in Dental Practice', which looked at the implications of new restorative materials for the future practice of dentistry, was published in the *British Dental Journal*.^52^ Elderton^53^ discussed how traditional cavity designs for Class II lesions based on the Black^54^ ‘extension for prevention' mantra was responsible for ‘much of the over-zealous removal of healthy tooth tissue that is still commonplace today'.^53^ Elderton advocated maximally conservative preparation of the tooth to maintain the tooth strength and stressed that if a small restoration failed that there should be sufficient tooth tissue available for a replacement restoration.^53^ David Brown^55^ concluded that the replacement of dental amalgam as the restorative material of choice would not occur ‘overnight, as 150 years' of experience is a lot to catch up on.' McLean observed that 30 years of composite research had not improved the fracture toughness, wear performance or adhesiveness of the low-viscosity resin without the addition of diluents to enable incorporation of fillers without increased polymerisation shrinkage.^56^ While the trend towards microfine filler particles had been accelerated, through the use of colloidal silica to improve polishability, most studies at the time showed greater wear than did dental amalgam.^56^ The author also advocated the use of cermet cements, which were at the developmental stage, although clinical experience over four years in Class II cavities were ‘worthy of further investigation'.^56^ Yardley^57^ discussed the possibility of glass-ionomer cements as an alternative to dental amalgam owing to the chemical attachment of the poly(acrylic) acid to tooth structure as the mechanism of a truly adhesive restorative. McCabe^58^ discussed the complications of employing light-activated materials, including the problems that most such manufacturers recommend a specific light source, and that the degree of polymerisation was not uniform throughout compared with ‘chemically cured' composite, while formulation influenced the depth of cure. Additionally, the position of the light source (angulation, proximity to the tooth, given the ease of access and cavity shape) influenced the depth of cure along with the intensity of the light source where ‘doubling the exposure time does not double the depth of cure'.^58^ The ‘race' among dental resin composite manufacturers' to ‘produce the greatest depth of cure with the shortest possible exposure time takes no account of the practical difficulties and could be doing a disservice to the patient, dentist and manufacturer' was also discussed.^58^ McCabe^58^ further predicted that improvements in composite resin formulations were ‘unlikely while manufacturers simply ring the changes on filler size and make minor alterations to resin composition.' McCabe was depressingly accurate when he concluded that, ‘it is a safe prediction that within a few years there will be as many different bonding systems available as there are different types of composite – happy days!'^58^

Three years earlier an alternative narrative from the USA came from a ‘symposium on composite resins in dentistry' published in 1981.^59^ Harold Horn in his ‘foreword' stated:

‘Lastly, the true pioneers who are actually responsible for the progress in restorative resin technology may receive less than appropriate recognition. Indeed, they are the ones that should take a bow. Dentists and patients are indebted to Dr. Ray L. Bowen for developing the Bis-GMA, to Dr. Michael G. Buonocore for introducing acid-etching, and to pioneers in dental resin research such as Dr. Ralph W. Phillips and Dr. Robert G. Craig,'^59^ highlighting a lack of recognition, participation and appreciation of the UK effort. At that meeting, Phillips^60^ said:

‘*At the outset it is important to emphasise that the resin restoration is a demanding one and is not easily mastered. Too frequently the dentist is led to believe that because of the apparent simplicity of placement, resin is a somewhat foolproof material. Nothing is further from the truth*.'

While looking to the future he anticipated the development of ‘adhesive, wear resistant dental polymers' but advocated long-term clinical studies.^60^ Leinfelder was adamant that the resin materials available could not ideally be a replacement for dental amalgam and ‘should not be recommended for the restoration of class II cavity preparations'.^61^ Throughout the 1980s, review articles for RBCs^44^^,^^45^^,^^47^^,^^48^^,^^49^^,^^50^^,^^51^ were commonplace but contributions by Roulet were of particular significance to the dental materials scientist^62^ and dental practitioner,^63^ pointing out that since RBCs must compete with dental amalgam in occlusal load-bearing molar teeth, then both ‘wear resistance must be equivalent to dental amalgam'^62^ and an ‘impermeable marginal seal and ideal marginal integrity'^62^ are required throughout service. However, he also noted that wear resistance was inconsistent to measure clinically and that marginal integrity was virtually impossible to determine chairside, highlighting the difficulties with a controlled clinical trial where these were the requirements.

## The future of restorative dentistry

When looking to the future of restorative dentistry in 1978, McLean suggested that, despite the significant advances in RBC development over the previous 30 years, ‘there are still only two really permanent materials, gold and porcelain'.^12^ Later, in 1980, he observed that:

*‘The future of aesthetic restorative dentistry lies in developing new technologies rather than the simple substitution of one material for another. Research in the polymer chemistry and metallurgical fields have reached a plateau and the dental profession would be well advised not to adopt a Panglossian attitude to the development of new materials*'.^6^

A Panglossian attitude (being ‘excessively optimistic') has engulfed dentistry and is not confined to glossy manufacturers' brochures. Dental resin-based composites based on oxirane and silorane monomeric resin constituents^64^ were novel and based on a ring-opening-polymerisation in an effort to reduce polymerisation shrinkage (and associated consequences) but were withdrawn from the market soon after their introduction. Otherwise, improvements in RBCs were based on the simple substitution of one material for another^6^ or ‘manufacturers simply ring the changes on filler size and make minor alterations to resin composition'^58^ resulting in so-called ‘packable',^65^ ‘flowable' and ‘bulk fill flowable' materials,^66^ with ‘bulk fill' restorative^67^ and ‘universal' products^67^ emphasising the Panglossian attitude to RBC development.^6^ Have high-intensity light irradiation technologies^68^^,^^69^ and bulk fill materials^66^^,^^67^ been introduced to reduce clinical placement times rather than offer actual clinical improvements? Is this not just dumbing down clinical skills?

## Conclusion

To John Walford McLean, I give the last word. In 1978 he postulated that ‘advances in science must be viewed in decades rather than years and certainly the last two decades in restorative dentistry have been of great significance'.^12^ Depressingly, the same cannot be said about the last three or four decades (1990–2026) in terms of resin based composite development for tooth tissue-mimicking restorative dentistry. Where are the innovators to make up for those lost decades?
